# Self-Experienced Cognitive Function in the Digital Era: Are Older Adults at Risk of Subjective Cognitive Decline?

**DOI:** 10.1093/geroni/igab046.2775

**Published:** 2021-12-17

**Authors:** Samantha Smith, Moriah Splonskowski, Claudia Jacova

**Affiliations:** 1 Pacific University, Hillsboro, Oregon, United States; 2 Pacific University, Forest Grove, Oregon, United States

## Abstract

Older adults often find it difficult to use everyday technology proficiently. We hypothesized that these difficulties would be exacerbated in those with subjective cognitive decline (SCD), that is, self-perceived worsening of cognitive functions that has been associated with increased risk of future dementia. Here we investigated the relationship between SCD symptom burden and technology proficiency. A nation-wide sample of adults (N=483) ages 50-79 (66.5% female; 14.5% age >70) completed an online survey via a crowdsourcing website, Amazon Mechanical Turk. The survey included the Subjective Cognitive Decline Questionnaire (SCD-Q MyCog) (0-25, M=4.71, SD=5.77), questions about respondents’ proficiency with computer, smartphone, and tablet (4-12, M=9.72, SD=1.97), and the PROMIS depression (M=13.18, SD=6.32) and anxiety (M=13.04, SD=5.68) scales. Linear regression was used to examine the ability of technology proficiency to predict SCD score. We also probed the interaction of technology proficiency with age ( <70 vs. >70 years), and adjusted for covariates. We found that the age/technology interaction (B=-0.80), older age (B=7.49), lower education (B=-1.08), higher depression (B=0.20) and anxiety (B=0.16) symptoms predicted higher SCD burden (R-squared=.16). For respondents >70 years low technology proficiency predicted high SCD burden (B=-.79) whereas for those <70 years no relationship was found. Our study draws attention to older adults’ self-experienced cognitive function in the digital era. The association between low technology proficiency and SCD may signal the adverse impact of the digital era on those who experienced technology only later in life. It is equally possible that declining technology proficiency is an indicator of emerging neurodegenerative disease.

